# Demographic effects of a megafire on a declining prairie grouse in the mixed‐grass prairie

**DOI:** 10.1002/ece3.9544

**Published:** 2022-11-30

**Authors:** Nicholas J. Parker, Daniel S. Sullins, David A. Haukos, Kent A. Fricke, Christian A. Hagen, Adam A. Ahlers

**Affiliations:** ^1^ Department of Horticulture and Natural Resources Kansas State University Manhattan Kansas USA; ^2^ U.S. Geological Survey, Kansas Cooperative Fish and Wildlife Research Unit, Division of Biology Kansas State University Manhattan Kansas USA; ^3^ Kansas Department of Wildlife and Parks Emporia Kansas USA; ^4^ Department of Fisheries, Wildlife, and Conservation Sciences Oregon State University Corvallis Oregon USA

**Keywords:** chick survival, demography, lesser prairie‐chicken, nest survival, *Tympanuchus pallidicintus*, wildfire

## Abstract

Recent studies have documented benefits of small, prescribed fire and wildfire for grassland‐dependent wildlife, such as lesser prairie‐chickens (*Tympanuchus pallidicintus*), but wildlife demographic response to the scale and intensity of megafire (wildfire >40,000 ha) in modern, fragmented grasslands remains unknown. Limited available grassland habitat makes it imperative to understand if increasing frequency of megafires could further reduce already declining lesser prairie‐chicken populations, or if historical evolutionary interactions with fire make lesser prairie‐chickens resilient. To evaluate lesser prairie‐chicken demographic response to megafires, we compared lek counts, nest density, and survival rates of adults, nests, and chicks before (2014–2016) and after (2018–2020) a 2017 megafire in the mixed‐grass prairie of Kansas, USA (Starbuck fire ~254,000 ha). There was a 67% decline in attending males on leks post‐fire and a 57% decline in occupied leks post‐fire. Despite population declines as indicated by lek counts, adult female breeding season survival ($\hat{S}$) was similar pre‐ ($\hat{S}$ = 0.65 ± 0.08 [SE]) and post‐fire (0.61 ± 0.08), as was chick survival (pre‐fire: 0.23 ± 0.07; post‐fire: 0.27 ± 0.11). Nest survival appeared lower post‐fire (pre‐fire: 0.38 ± 0.06; post‐fire: 0.20 ± 0.06), but did not differ at the 95% confidence interval. Nest density of marked females declined 73% in areas burned by megafire. Although lesser prairie‐chickens persisted in the study area and we documented minimal effects on most demographic rates, reduced lesser prairie‐chicken abundance and reproductive output suggests full recovery may take >3 years. Increased propensity for megafire resulting from suppression of smaller fires, compounded by climate change and woody encroachment, may impose a short‐term (3–5 year) threat to already declining lesser prairie‐chicken populations.

## Graphical abstract

Megafires are increasing globally and in the Great Plains, USA, potentially threatening grassland dependent wildlife in the region. We examined short‐term lesser prairie‐chicken demographic response to megafire and found minimal impacts to vital rates; however, reproductive output and the overall population declined. While evolutionary interaction with fire likely provided lesser prairie‐chickens some resilience to megafire, increased megafire potential still poses a threat to this declining species.

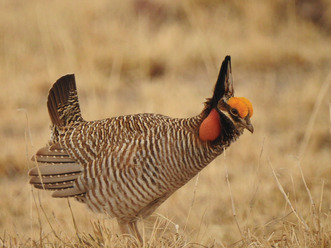

## 1 INTRODUCTION

Increases in the frequency and magnitude of massive wildfires, known as megafires, are a predicted result of climate change (Barbero et al., 2015; Cao et al., 2015; Stephens et al., 2014). While exact definitions vary, megafires are generally defined as a wildfire covering >10,000–40,000 ha (Lindley et al., 2019; Linley et al., 2022; USFS, 2018) and are characterized by large social and economic effects (Stephens et al., 2014; Williams et al., 2011). Unfortunately, effects of megafire on wildlife are difficult to quantify due to the limited opportunity for planning before‐and‐after‐impact studies. Current research on wildlife response to megafire is mixed and highly context‐dependent, with some wildlife appearing resilient to megafire impacts (Kreling et al., 2021; Mirts et al., 2022; Santos et al., 2022; Siegel et al., 2019). Conversely, some threatened species, such as spotted owl (*Strix occidentalis*; Jones et al., 2016, 2021; Stephens et al., 2014) and greater sage‐grouse (*Centrocercus urophasianus*; Anthony et al., 2022; Dudley et al., 2021; Foster et al., 2019), have lost habitat and exhibited population declines as a result of megafire. Even fire‐adapted species that benefit from smaller wildfires, such as black‐backed woodpecker (*Picoides arcticus*), can be negatively affected by megafires that burn large areas and reduce access to mosaics of burned and unburned patches that provide critical resources (Stillman et al., 2019).

The lesser prairie‐chicken (*Tympanuchus pallidicintus*), a declining prairie grouse species endemic to the southern Great Plains, has experienced multiple megafires throughout its distribution in recent years (Donovan et al., 2017; Hagen & Giesen, 2005; Lindley et al., 2019). Lesser prairie‐chickens currently occupy only 14% of their estimated historic range and rely heavily on the few remaining grassland‐dominated landscapes within their distribution (Garton et al., 2016; Haukos & Zaveleta, 2016). Habitat quality in remaining grasslands has been degraded by alteration in frequency and intensity of fire, grazing, drought, and invasion of woody plants (Fuhlendorf et al., 2017; Fuhlendorf & Engle, 2001; Haukos & Zaveleta, 2016). As a result, lesser prairie‐chickens were listed as threatened in 2014 under the 1973 Endangered Species Act. Despite the listing status being rescinded in 2016, a 2021 proposed listing indicates the lesser prairie‐chicken remains a species of concern requiring conservation efforts (USFWS, 2014, 2021). Identified within this proposed listing is the potential threat posed to lesser prairie‐chickens from the increased area and severity of wildfires throughout their distribution, highlighting the need for knowledge regarding lesser prairie‐chicken response to megafires (USFWS, 2021).

Frequent fire disturbance helped shape grassland systems in the Great Plains and play an important role in lesser prairie‐chicken ecology (Axelrod, 1985; Fuhlendorf & Engle, 2001; Samson et al., 2004). An estimated fire return interval of 4–10 years in the southern mixed‐grass prairie promoted biodiversity, vegetation heterogeneity, and prevented woody species establishment in grasslands (Axelrod, 1985; Brockway et al., 2002; Wright & Bailey, 1982). Selective grazing by bison (*Bison bison*) in recently burned areas helped create a shifting mosaic of habitat types, with recently burned and grazed areas of short vegetation in close proximity to thicker, dense vegetation not recently burned (Askins et al., 2007; Fuhlendorf & Engle, 2001). Having evolved under such a paradigm, lesser prairie‐chickens require grasslands heterogeneous in herbaceous cover, vertical structure, and composition for different habitat needs (Askins et al., 2007; Haukos & Zaveleta, 2016). Quality forage, brood, and lek (communal courtship area) habitat occur in recently burned areas, but lesser prairie‐chickens also need denser, unburned vegetation for nest and adult cover (Boyd & Bidwell, 2001; Cannon & Knopf, 1979; Haukos & Zaveleta, 2016; Lautenbach et al., 2021). While grassland heterogeneity was previously maintained by fire, grazing, and climatic influences, European settlement altered these drivers, leading to homogenous grasslands and degrading lesser prairie‐chicken habitat (Fuhlendorf & Engle, 2001). As lesser prairie‐chickens avoid trees and other tall features, woody encroachment due to fire suppression has reduced and fragmented lesser prairie‐chicken habitat (Fuhlendorf et al., 2017; Lautenbach et al., 2017). Prescribed burns and patch‐burn grazing systems can benefit lesser prairie‐chickens by restoring grasslands and creating small‐scale heterogeneity in vegetation structure (Boyd & Bidwell, 2001; Cannon & Knopf, 1979; Jones, 2009; Lautenbach et al., 2021); however, the effects of large‐scale and intense megafires on lesser prairie‐chicken populations in modern, altered grasslands are largely unknown.

Demographic rates of lesser prairie‐chickens can be influenced by disturbances such as anthropogenic expansion, landscape fragmentation, grazing, and fire (Kraft et al., 2021; Lawrence et al., 2021; Robinson, Haukos, Plumb, Kraft, et al., 2018). Intensive grazing of grasslands can reduce herbaceous cover and decrease lesser prairie‐chicken nest survival rates (Kraft et al., 2021). Reduced adult and nest survival have been documented for greater prairie‐chickens (*Tympanuchus cupido*) occurring in areas with annual intensive burning and grazing practices, compared to patch‐burn grazing systems that mimic historic disturbance regimes (McNew et al., 2015; Winder et al., 2018). A megafire may mimic annual intensive burning and grazing over a larger area, reducing available habitat for lesser prairie‐chickens at a landscape scale. Alternatively, megafire could have limited impacts or improve habitat if grassland recovery is similar to small‐scale fire, and post‐fire grazing and weather conditions align to allow increased primary productivity and biodiversity (Collins & Barber, 1986; Gates et al., 2017; Vermeire et al., 2014). Brooding is one life stage that is most likely to benefit from recent fire. Increased chick survival has been linked to increased availability of forbs on the landscape (Fields et al., 2006), suggesting megafire may improve chick survival if it increases forbs and open space on the landscape similar to grassland response to prescribed fire (Boyd & Bidwell, 2001; Lautenbach et al., 2021; Patten et al., 2007). Chick survival is essential for maintaining lesser prairie‐chicken populations and could drive population responses to megafire (Hagen et al., 2009; Ross et al., 2018; Sullins, Kraft, et al., 2018). Evaluating demographic rates across multiple life stages is key to understanding lesser prairie‐chicken population recovery following megafire. Localized population recovery may be driven by demographic rates because there is limited movement among lesser prairie‐chicken populations, making immigration and emigration relatively minor factors in post‐fire population persistence (DeYoung & Williford, 2016). Restriction of lesser prairie‐chicken populations to limited, isolated grassland patches make them particularly susceptible to extirpation from stochastic events such as megafire (DeYoung & Williford, 2016; Fuhlendorf et al., 2002; Simberloff, 1994), underscoring the importance of evaluating short‐term demographic responses of lesser prairie‐chickens to megafire.

Our objective was to assess megafire's potential effects on a lesser prairie‐chicken population in the Mixed‐Grass Prairie Ecoregion (McDonald et al., 2014), a region that has experienced increased wildfire activity (Donovan et al., 2017; Lindley et al., 2019) and where lesser prairie‐chicken populations have declined an estimated 60% since 2012 (Nasman et al., 2021). We evaluated the influence of a 2017 megafire (Starbuck fire, ~254,000 ha) on multiple lesser prairie‐chicken demographic parameters using a before‐and‐after‐impact design. Using data collected before (2014–2016) and after (2018–2020) the 2017 megafire, we quantified differences in pre‐ and post‐fire lek counts and adult, nest, and chick survival rates. We predicted chick survival would increase post‐fire, as the fire could have created areas of quality brood habitat (i.e., increased forbs and open areas). We further hypothesized the megafire would reduce lesser prairie‐chicken abundance in the area, as we predicted effects of the megafire on cover and habitat would lower adult and nest survival.

## 2 MATERIALS AND METHODS

### 2.1 Study area

**FIGURE 1:**
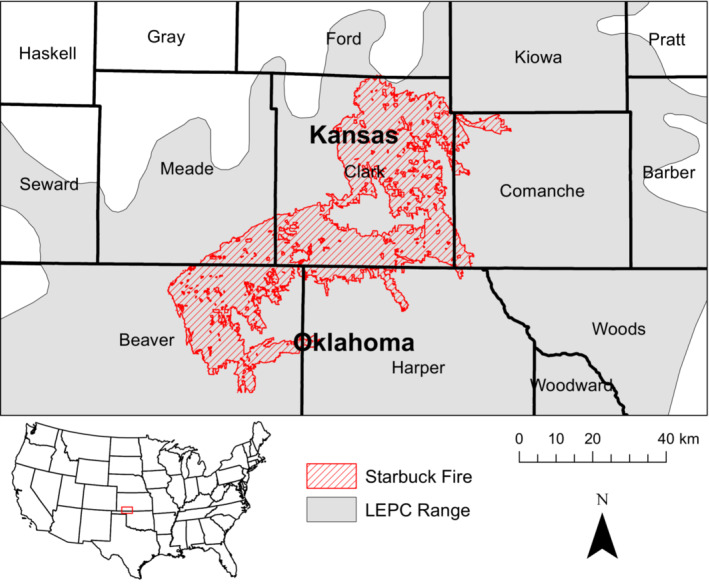
Map of the study area in Clark County, Kansas, USA, detailing the extent of the 2017 Starbuck fire in Kansas and Oklahoma in the Mixed‐Grass Prairie Ecoregion of the lesser prairie‐chicken range.

The Starbuck fire burned approximately 253,810 ha (627,178 acres) in Kansas and Oklahoma, USA, from 6–7 March 2017 (Kansas Forest Service, 2019), becoming the largest fire in recorded Kansas history (Lindley et al., 2019; Sirch et al., 2022). The Starbuck fire caused an estimated $44 million (USD) in damage to fencing and structures and killed 5000–9000 cattle (*Bos taurus*; Bickel, 2018; Rethorst et al., 2018). The spatial extent of the Starbuck fire was entirely within the Mixed‐Grass Prairie Ecoregion, one of four ecoregions occupied by lesser prairie‐chickens (Figure 1; McDonald et al., 2014). The Mixed‐Grass Prairie Ecoregion contained some of the largest tracts of contiguous grassland and available habitat throughout the lesser prairie‐chicken range (Spencer et al., 2017; Sullins et al., 2019) and was estimated to support 10% of the entire lesser prairie‐chicken population in 2021 (Nasman et al., 2021).

Our study was conducted in Clark County, Kansas, on the western edge of the mixed‐grass prairie. The majority of the area was managed for livestock production, with no use of prescribed fire. A low‐intensity, long‐duration rotational grazing system among large pastures was used for both cow/calf and yearling herds, targeted to remove half of the available forage each growing season. Following the loss of many cattle in the fire, grazing pressure varied post‐fire as ranches recovered and restocked. Other land use included row‐crop agriculture, energy extraction, and properties enrolled in the U.S. Department of Agriculture Conservation Reserve Program (CRP). The area was composed of 76.6% native working grassland, 14.2% cropland, and 5.5% CRP (Robinson, Haukos, Plumb, Kraft, et al., 2018). We define native working grasslands as grasslands that are typically grazed and used for livestock production. Tracts of CRP in the study area were typically planted with native mixed and tall‐grass plant species. Many CRP fields did not burn as they were surrounded by a matrix of cropland, which provided a protective buffer of bare ground or green crop at the time of the fire (Donovan et al., 2020).

Average annual temperatures ranged from 6.1 to 21.2°C, with a 50‐year (1970–2020) average annual precipitation of 58.3 cm (NOAA, 2020). The majority of rainfall is received in the spring and early summer (Apr–Jul). Average annual precipitation was similar before (2014–2015; 69.7 cm) and after (2018–2019; 71.6 cm) the megafire (NOAA, 2020, Table S1). Major soil types were fine loamy sands, fine sandy loams, and fine sands (Soil Survey Staff, 2020), and common plant species included sand dropseed (*Sporobolus cryptandrus*), western ragweed (*Ambrosia psilostachya*), little bluestem (*Schizacyrim scoparium*), Russian thistle (*Salsola tragus*), alkali sacaton (*Sporobolus airoides*), sand sagebrush (*Artemisia filifolia*), and blue grama (*Bouteloua gracilis*; Parker et al., 2022).

### 2.2 Lek counts

We counted male lesser prairie‐chicken attendance at leks before and after megafire as an index of changes in population abundance and distribution across our study area. We visited the same lek locations before and after the fire and each year surveyed the surrounding area for new leks. Lek surveys occurred during the peak lekking period from 15 March–1 May pre‐ (2014, 2015) and post‐fire (2018, 2019). Each known lek was surveyed at least twice during this period. As leks are known to be dynamic (Hovick, Allred, et al., 2015), leks that moved relatively short distances (<1 km) were not counted as new leks. We surveyed extensively for new leks using auditory surveys and searching for displaying males within the study area and nearby grassland areas where we had permission. We surveyed between sunrise and 1000 during favorable weather conditions (wind <24 km/h) to increase likelihood of detecting all individuals. Prior to cessation of displays at each lek, we flushed birds to ensure count accuracy and recorded seasonal high counts of male attendance.

### 2.3 Capture

We captured lesser prairie‐chickens on leks in the spring of 2014 and 2015 before the fire, and in the spring of 2018 and 2019 after the fire, using walk‐in funnel traps and tension drop nets (Haukos et al., 1990; Silvy et al., 1990). We sexed captured birds based on pinnae length and tail feather coloration (Copelin, 1963). We aged birds as either second‐year (SY) or after‐second‐year (ASY) based on wear and coloration of primary flight feathers (Ammann, 1944). All captured females were banded and given either a 22‐g Argos Satellite PTT transmitter (SAT‐PTT; PTT‐100, Microwave Technology) or a 15‐g bib style VHF transmitter (Advanced Telemetry Systems; Kraft et al., 2021; Robinson, Haukos, Plumb, Lautenbach, et al., 2018). We marked all captured females with transmitters because we were primarily interested in female survival and reproductive success as these contribute most to overall demographic rates of populations. All methods were approved by the Kansas State University Institutional Animal Care and Use Committee (protocol numbers 3241, 3703, and 4193) and the Kansas Department of Wildlife and Parks (scientific collection permit numbers SC‐079‐2014, SC‐001‐2015, SC‐024‐2018, and SC‐015‐2019).

### 2.4 Adult survival

To assess adult female survival, we located VHF‐radiomarked lesser prairie‐chickens 2–3 times per week via triangulation using a 3‐element handheld Yagi antenna and radio receiver (Advanced Telemetry Systems, and Communication Specialists, Inc.). Satellite transmitters provided locations every two hours from 0400–2200 resulting in ~8–10 locations per day. Locations were uploaded to the Argos satellite system every three days and downloaded weekly. We searched for, and investigated, mortalities as soon as possible following activation of a mortality switch for VHF transmitters (within two days) and activity sensor data for satellite transmitters (within seven days). At each mortality location, we classified cause of death as mammalian predator, avian predator, or unknown (Hagen et al., 2007; Appendix S1).

We used known‐fate Kaplan–Meier models to estimate weekly adult female lesser prairie‐chicken survival pre‐ and post‐fire in the R statistical environment (R Core Development Team, 2021) using the “survival” package (Therneau, 2015). We estimated overall survival separately for the 27‐week breeding season (15 Mar–15 Sep) and 25‐week nonbreeding season (16 Sep–14 Mar). We did not consider survival estimates with overlapping 95% confidence intervals as statistically different.

While Kaplan–Meier models provide reliable and easy‐to‐interpret estimates of survival, they are limited in their ability to test the influence of variables on survival and compare models (Robinson, Haukos, Plumb, Lautenbach, et al., 2018; Winder et al., 2014). Therefore, we used Cox proportional hazard models in R (package “survival”) to test for the effects of several covariates on adult female survival during the breeding season (Fox & Weisberg, 2011; Therneau, 2015). We restricted covariate modeling with Cox proportional hazards models to the breeding season as survival during this time can be more important for population persistence and sample sizes facilitated inclusion of more parameters (Hagen et al., 2009; Ross et al., 2018). We tested univariate models of year, fire status, and transmitter, as well as additive and interactive combinations of these variables. Year was a categorical variable that tested for differences in breeding season survival for each year of the study. Fire status tested for a difference in breeding season survival before and after the megafire. Although transmitter type is not known to influence lesser prairie‐chicken survival, it may affect survival for other grouse in post‐fire landscapes; therefore, we included transmitter type as a covariate, testing for differences in survival between females marked with VHF and SAT‐PTT transmitters (Foster et al., 2018; Lawrence et al., 2021). We ranked models using Akaike's Information Criterion corrected for small sample size (AICc) and considered models ΔAICc ≤ 2 competitive (Burnham & Anderson, 2002).

### 2.5 Nest survival and density

We closely monitored marked hens for nesting behavior and identified nesting hens based on females remaining at one location for >3 days (Lautenbach et al., 2019). We monitored nests following McNew et al. (2009) and Lautenbach et al. (2019) and returned to estimate nest fate once the hen was off nest for >1 day or killed. We considered nests successful if ≥1 egg hatched, based on pipped eggshells. If eggs were intact, the nest was considered abandoned. For unsuccessful nests, we examined remaining eggshells and nest bowls for signs of trampling or nest predators. We broadly classified nest predators as mammalian, snake, or unknown following Sargeant et al. (1998) and Pitman et al. (2006; Appendix S1).

To evaluate if megafire affected fine‐scale vegetation characteristics important to lesser prairie‐chicken nest survival, we measured vegetation at each nest site within three days of nest fate following standardized protocols (Lautenbach et al., 2019; Parker et al., 2022). At each nest, we measured vegetation height and estimated percent cover of grasses, forbs, shrubs, litter, and bare ground using a modified 60 × 60 cm Daubenmire frame at the nest bowl and locations 4 m from the nest bowl in each cardinal direction (Daubenmire, 1959). We estimated height of 100%, 75%, 50%, 25%, and 0% visual obstruction using a Robel pole from each cardinal direction 4 m away from the nest bowl at a height of 1 m (Robel et al., 1970). We included different percentages of visual obstruction as each correlates with measures of vegetation thickness and structure that are important for the various habitat needs of lesser prairie‐chickens (Gehrt et al., 2020; Lautenbach et al., 2019, 2021; Parker et al., 2022). We measured litter depths every 0.5 m along 4 m transects in each cardinal direction away from the nest bowl. For subsequent analysis, we averaged across all readings for each vegetation measurement at the nest.

We estimated daily nest survival rates using the nest survival procedure in the RMark package in R (Dinsmore et al., 2002; Laake, 2013). We used a 28‐day exposure period for the average incubation period of 28 days to estimate nest survival, then applied the delta method to calculate variance around the estimate (Grisham et al., 2014; Lautenbach et al., 2019; Powell, 2007). We fit candidate models for nest survival using a priori selected variables and ranked them using AICc (Burnham & Anderson, 2002). We took a hierarchical modeling approach, with our first model set (*n* = 19 models) testing single variable and quadratic vegetation characteristic models identified as important in previous work (Fields et al., 2006; Lautenbach et al., 2019). The top model from the vegetation model set was included in the final model set (*n* = 32 models). A subsample of nests lacked vegetation data (18%), so to use the full dataset for the final model set, we used mean vegetation values for nests missing data (Grisham et al., 2014; Lautenbach et al., 2019). The final model set included variables known to influence lesser prairie‐chicken nest success (nesting attempt [first or renest] and hen age [second year or after second year]), variables related to the megafire (year, fire status, burned area, actually burned, and cover type), and additive and interactive combinations of these variables. Fire status simply specified whether the nest was pre‐ or post‐fire. Burned area specified if the nest was inside the fire perimeter or not, regardless of the fire status. Actually burned was similar, but compared only post‐fire nests inside the burn perimeter to all other nests. Cover type evaluated nest survival in different land cover types (delineated as described below), which included native working grassland (grasslands managed for grazing), CRP, cropland, and other (all other cover types). We included a posteriori covariate models of precipitation received during the entire nesting season (Apr–Jul) and for the primary months of nesting (May–Jun) for each year after experiencing severe weather that we suspected to have a major effect on survival (Londe et al., 2021; NOAA, 2020; Table S1). Models with ΔAICc ≤2 were considered competitive.

While individual nest survival provides one metric to assess megafire effects on reproductive output, we were interested in determining if megafire altered the density and placement of nests on the landscape, thereby affecting reproductive output at the population level (Van Horne, 1983). To evaluate reproductive influences at the population level, we estimated relative nest densities based on the number of nests from marked lesser prairie‐chickens before and after the megafire (Pidgeon et al., 2006; Sullins, Kraft, et al., 2018). We calculated nest densities within a 5‐km radius surrounding each active lek, then averaged among leks. Within these 5‐km buffers, we estimated overall nest densities, nest densities in burned and unburned areas, and nest densities in native working grasslands (grasslands managed for grazing) and CRP fields (Sullins, Kraft, et al., 2018). We compared nest densities before and after the fire and between land cover types using Mann–Whitney *U*‐tests in R (*α* = .05). We used the 2011 National Land Cover Database to identify all areas of grassland and within those a 2014 CRP layer provided by the USDA Farm Service Agency to identify CRP fields. Areas burned by the Starbuck fire were identified using spatial layers compiled by the Monitoring Trends in Burn Severity program (MTBS, 2019).

### 2.6 Chick survival

Once we identified a successful nest, we performed weekly flushes of the female and her brood to estimate chick survival. We flushed broods at or before sunrise each week at approximately 7, 14, 21, 28, and 35 days post‐hatch, dependent on calm weather conditions and availability of a GPS point from satellite transmitters. We counted the number of chicks accompanying the female and recorded their location during each flush. If we did not encounter chicks on two consecutive flushes, we identified the brood as unsuccessful. After 35 days, chick survival approximates adult survival and we stopped flushing broods (Hagen et al., 2009).

We used Lukacs' young survival from a marked adult model to estimate week‐specific chick survival in Program MARK (Lukacs et al., 2004; White & Burnham, 1999). To determine if megafire influenced survival, we fit models of constant survival, constant survival before/after the fire, week‐specific survival, and week‐specific survival before/after the fire. Models were ranked using AICc and models with ΔAICc ≤2 were considered competitive (Burnham & Anderson, 2002). Small sample sizes precluded extensive modeling of survival and detection probability, so we held detection constant as methodology did not change before and after the fire. We derived 35‐day survival estimates before and after the fire as a product of weekly estimates with error calculated using the delta method assuming independence (Powell, 2007).

## 3 RESULTS

### 3.1 Lek counts

The average high count of males across all leks surveyed decreased 67% after the wildfire (Table 1). Average high count of males per lek declined from 10.74 ± 6.51 $\left(\bar{x}\pm \mathrm{SD}\right)$ before to 3.17 ± 5.06 after (occupied and unoccupied leks combined; Table 1). At occupied leks post‐fire, the average male high count was 6.79 ± 5.54 males. From the 14 active leks in 2015, six were vacated in 2018, and two additional leks were unoccupied in 2019, representing a 57% decrease in the total number of occupied leks (Table S2). No new leks were found post‐fire within the surveyed area, although some moved short distances (<1 km).

**TABLE 1:** Average male high counts per lek, standard deviation (SD), and summed high counts of male attendance across leks in Clark County, Kansas, USA, surveyed before (2014–2015) and after (2018–2019) the Starbuck fire in March 2017.

Year	Average males/Lek	SD	Total count
Before			
2014	10.67	6.51	128
2015	10.80	6.73	162
Average	10.74	6.51	145
After			
2018	3.47	5.50	52
2019	2.87	4.75	43
Average	3.17	5.06	47.5

*Note*: 12 leks were surveyed in 2014, and 15 leks were surveyed each year after. Leks were surveyed from 15 Mar–1 May before 1000 and under favorable weather conditions.

### 3.2 Capture

Prior to the fire, we marked 46 females (26 in 2014, 20 in 2015) with VHF (*n* = 18) or GPS transmitters (*n* = 28). Post‐fire, we marked 31 females (9 in 2018, 22 in 2019) with transmitters (VHF = 4, GPS = 27). The majority were second‐year females both before (*n* = 34) and after (*n* = 25) the fire.

### 3.3 Adult survival

**FIGURE 2:**
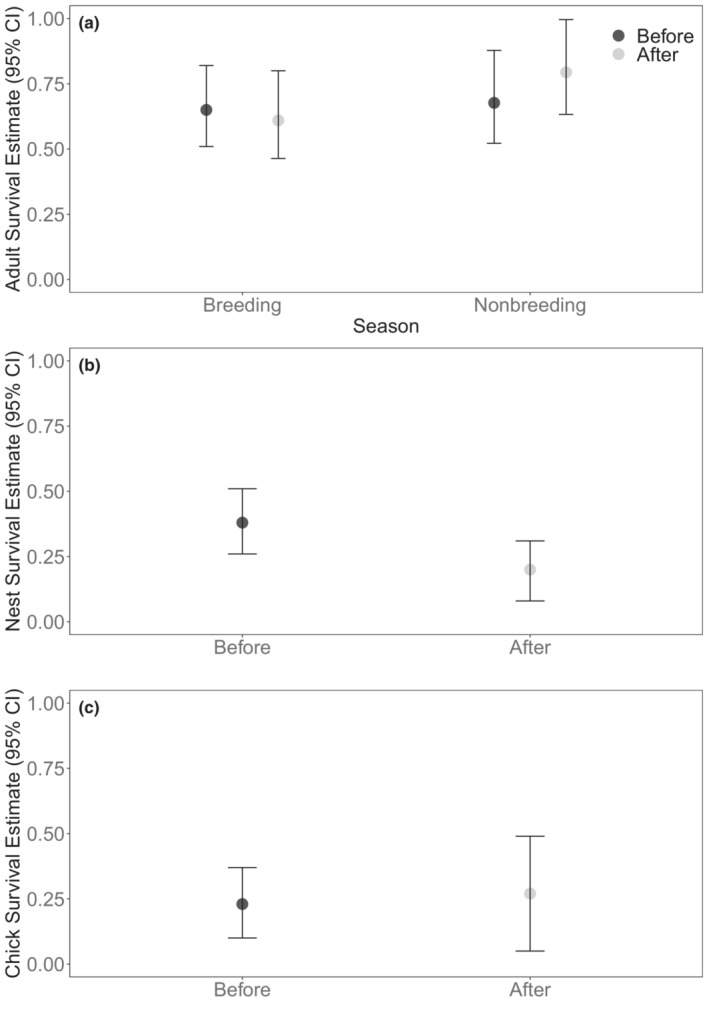
Survival estimates and 95% confidence intervals from Kaplan–Meier survival models of female adult lesser prairie‐chickens in the breeding (15 Mar–15 Sep) and nonbreeding (16 Sep–14 Mar) seasons (a); nest survival models for lesser prairie‐chicken nests over a 28‐day exposure period (b); Lukacs' young survival models of lesser prairie‐chicken chicks over 35‐days (c) in Clark County, Kansas, USA, before (2014–2016) and after (2018–2020) the 2017 Starbuck fire.

Breeding season and nonbreeding season survival rates of female lesser prairie‐chickens were similar before and after the megafire (Figure 2a). Breeding season survival ($\hat{S}$) for female lesser prairie‐chickens pre‐fire was 0.65 ± 0.08 ($\hat{S}$ ± SE, 95% CI = 0.51–0.82), and post‐fire was 0.61 ± 0.08 (95% CI = 0.46–0.80). For breeding season survival, the null model was the top‐ranked model (*w*_*i*_ = 0.48, Table 2), followed by single variable models for fire status (ΔAICc = 1.80, *w*_*i*_ = 0.20, *β*_fire_ = −0.19, SE = 0.37, 95% CI = −0.92–0.55) and transmitter (ΔAICc = 1.88, *w*_*i*_ = 0.19; *β*_transmitter_ = −0.18, SE = 0.46, 95% CI = −1.08–0.72). Based on the high ranking of the null model, and beta coefficients overlapping zero at the 95% confidence interval in other competitive models, we concluded that fire and transmitter type were spurious variables that did not influence adult female survival in the breeding season. Nonbreeding season survival for females was 0.68 ± 0.09 (95% CI = 0.50–0.85) before the fire and increased slightly post‐fire (0.79 ± 0.09; 95% CI = 0.63–0.99), but 95% confidence intervals for these estimates overlapped (Figure 2a).

**TABLE 2:** Model selection results from Cox proportional hazards models of breeding season (15 Mar–15 Sep) female adult lesser prairie‐chicken survival before (2014–2015) and after (2018–2019) the March 2017 Starbuck fire in Clark County, Kansas, USA.

Model	K^a^	Δ AICc^b^	AICc^c^	*w*_*i*_ ^d^	Deviance^e^
Null	0	243.08	0.00	0.48	243.08
Fire	1	244.88	1.80	0.20	242.84
Transmitter	1	244.96	1.88	0.19	242.92
Transmitter + Fire	2	246.89	3.81	0.07	242.76
Transmitter × Fire	3	249.01	5.93	0.02	242.74
Year	3	249.06	5.98	0.02	242.78
Transmitter + Year	4	251.19	8.10	0.01	242.72
Transmitter × Year	7	257.42	14.34	0.00	242.06

*Note*: Tested models include null, fire (before, after), transmitter (VHF, SAT‐PTT), year (2014, 2015, 2018, 2019), and additive and interactive combinations of these variables.

^a^ Number of parameters.

^b^ Difference in Akaike's Information Criterion, corrected for small sample size.

^c^ Akaike's Information Criterion, corrected for small sample size.

^d^ Akaike weights.

^e^ Deviance or −2 × loglikelihood.

Probable causes of mortality in the breeding season were similar before and after the fire, split between mammalian and avian predators (Table 3). In the nonbreeding season, the majority (89%) of pre‐fire mortality events were attributed to mammals, while all post‐fire mortality events were attributed to avian predators.

**TABLE 3:** Cause‐specific mortality of adult female lesser prairie‐chickens (*n*, percentage) in Clark County, Kansas, USA, before (2014–2016) and after (2018–2020) the Starbuck fire in March 2017.

	Mammal	Avian	Unknown	Total
Breeding season				
Before (*n* = 56)	8 (50%)	7 (44%)	1 (6%)	16
After (*n* = 34)	4 (31%)	7 (54%)	2 (15%)	13
Nonbreeding season				
Before (*n* = 32)	8 (89%)	0 (0%)	1 (11%)	9
After (*n* = 20)	0 (0%)	4 (100%)	0 (0%)	4

*Note*: Sample sizes in the first column are the number of individuals monitored for that season. Breeding season was defined as 15 Mar–15 Sep, and nonbreeding season was 16 Sep–14 Mar.

### 3.4 Nest survival and density

A total of 52 nests were monitored prior to the fire (27 in 2014, 25 in 2015) with 42 first attempts and 10 renests documented. Pre‐fire, first nest attempts had a median incubation initiation date of 4 May, ranging from 26 April–22 May. Post‐fire, we monitored 35 nests (9 in 2018, 26 in 2019) with 6 renesting attempts. First nests after the fire had a median incubation initiation date of 11 May (range: 27 April–10 June). Before the fire, apparent nest success was 34.6%, another 34.6% of nests were depredated by mammals, 15.4% were depredated by snakes, with the remainder either experiencing hen mortality (3.8%), abandonment (3.8%), or unknown causes (7.7%; Table 4). After the fire, apparent nest success was 20%, while 17% of nests were depredated by mammals, 42.8% were depredated by snakes, 8.6% had hen mortality, and 11.4% were unknown. Average clutch size for first nests was similar before (11.03 ± 1.60; $\bar{x}\pm \mathrm{SD}$) and after (10.04 ± 2.40) the megafire. Hatch rates from successful first nests were also not different (before: 0.83 ± 0.16, after: 0.84 ± 0.17).

**TABLE 4:** Lesser prairie‐chicken nest fates (*n*, percentage) in Clark County, Kansas, USA, before (2014–2015) and after (2018–2019) the Starbuck fire in March 2017.

Year	Successful	Nest depredated		Hen depredated	Abandoned	Unknown	Total
		Mammal	Snake				
Before							
2014	7 (26%)	15 (56%)	3 (11%)	0 (0%)	0 (0%)	2 (7%)	27
2015	11 (44%)	3 (12%)	5 (20%)	2 (8%)	2 (8%)	2 (8%)	25
Total	18 (35%)	18 (35%)	8 (15%)	2 (4%)	2 (4%)	4 (8%)	52
After							
2018	1 (11%)	2 (22%)	4 (44%)	0 (0%)	0 (0%)	2 (22%)	9
2019	6 (23%)	4 (15%)	11 (42%)	3 (12%)	0 (0%)	2 (8%)	26
Total	7 (20%)	6 (17%)	15 (43%)	3 (9%)	0 (0%)	4 (11%)	35

**FIGURE 3:**
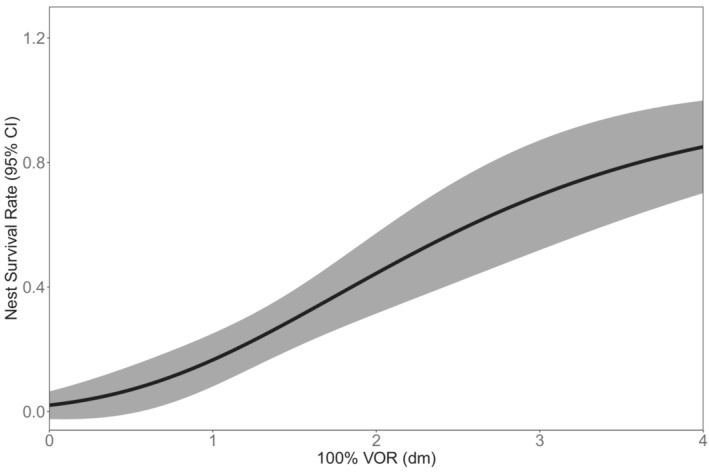
Lesser prairie‐chicken nest survival rate and 95% confidence interval (shaded area) over a 28‐day exposure period in relation to observed values of 100% visual obstruction (VOR) at nests from before (2014–2015) and after (2018–2019) the 2017 Starbuck fire in Clark County, Kansas, USA.

The single variable model of 100% visual obstruction was the top‐ranked model in our vegetation model suite (*w*_*i*_ = 0.24, Table S3). All other top‐ranked models with ΔAICc ≤ 2 were single variable models of visual obstruction from 75% to 25% and the quadratic effect of 100% visual obstruction. As all levels of visual obstruction were highly correlated, we included only 100% visual obstruction in the final model set. In the final model set, the model of 100% visual obstruction (*β*_VOR_ = .81, SE = 0.20, 95% CI = 0.41–1.21) alone was the best supported (*w*_*i*_ = 0.22, Table 5). Four other models had ΔAICc ≤ 2 and included 100% visual obstruction as one of multiple covariates (Table 5); however, beta coefficients for other covariates did not differ from zero and were not considered informative. Overall, 100% visual obstruction best‐predicted nest survival, with increased nest survival at higher levels of visual obstruction (Figure 3). While not a competitive model, we were interested in the fire's effects on survival. The single variable model of fire status indicated a decrease in nest survival post‐fire (*β*_fire_ = −.55, SE = 0.26, 95% CI = −1.06 to −0.03). Nest survival rates were 0.38 ± 0.06 (95% CI = 0.26–0.51) before the fire and 0.20 ± 0.06 (95% CI = 0.08–0.31) after the fire (Figure 2b).

**TABLE 5:** Model selection results of nest survival for lesser prairie‐chickens in Clark County, Kansas, USA.

Model	K^a^	Δ AICc^b^	AICc^c^	*w*_*i*_ ^d^	Deviance^e^
VOR	2	0.00	494.30	0.22	490.29
VOR + Attempt	3	0.27	494.57	0.19	488.55
VOR + BA	3	1.34	495.64	0.11	489.62
VOR + Fire	3	1.90	496.20	0.08	490.18
VOR + AB	3	1.98	496.28	0.08	490.26
VOR^2^	3	2.01	496.30	0.08	490.28
VOR × Attempt	4	2.07	496.37	0.08	488.34
VOR × BA	4	3.07	497.37	0.05	489.34
VOR + Fire + BA	4	3.35	497.65	0.04	489.62
VOR × AB	4	3.86	498.16	0.03	490.13
VOR × Fire	4	3.87	498.17	0.03	490.14
VOR × Fire × BA	8	9.27	503.57	0.00	487.47
Fire + Attempt	3	13.10	507.40	0.00	501.38
Fire	2	13.59	507.88	0.00	503.87
AB	2	13.95	508.24	0.00	504.24
${\text{Precip}}_{\text{April}–{\text{July}}^{2}}$	3	14.47	508.77	0.00	502.75
${\text{Precip}}_{\mathrm{May}–{\text{June}}^{2}}$	3	14.61	508.90	0.00	502.89
Fire + BA	3	14.96	509.26	0.00	503.24
Fire + Attempt + Precip_April–July_	4	15.07	509.37	0.00	501.34
Fire × Attempt	4	15.11	509.41	0.00	501.38
Attempt	2	15.48	509.78	0.00	505.77
Null	1	15.78	510.08	0.00	508.08
Precip_May–June_	2	15.94	510.24	0.00	506.23
Fire + Attempt + ${\text{Precip}}_{\text{April}–{\text{July}}^{2}}$	5	16.31	510.60	0.00	500.56
Year	4	16.48	510.78	0.00	502.75
Fire × BA	4	16.95	511.24	0.00	503.22
Age	2	16.96	511.26	0.00	507.25
Precip_April–July_	2	17.17	511.47	0.00	507.46
BA	2	17.66	511.95	0.00	507.94
Attempt × Age	4	18.13	512.43	0.00	504.40
Cover Type	3	18.65	512.95	0.00	506.93
Fire × Cover Type	5	19.53	513.82	0.00	503.78

*Note*: Models include single and quadratic variable combinations of 100% visual obstruction (VOR), fire (before, after), attempt (first, renest), burned area (BA; nest in fire perimeter), actually burned (AB, nest in burned area post‐fire), cover type (grassland, CRP, cropland, other), age (second year, after second year), year, total precipitation (Precip) during nesting periods (April–July, May–June), and null (intercept only).

^a^ Number of parameters.

^b^ Difference in Akaike's Information Criterion, corrected for small sample size.

^c^ Akaike's Information Criterion, corrected for small sample size.

^d^ Akaike weights.

^e^ Deviance or −2 × loglikelihood.

Overall nest density of marked females in the 5‐km‐radius surrounding leks before the fire was 0.74/10 km^2^ ± 0.09 $\left(\bar{x}\pm \mathrm{SE}\right)$, declining to 0.31/10 km^2^ ± 0.06 after the fire (Mann–Whitney *U*‐test = 217, *p* < .001). Pre‐fire, the majority (79%) of the landscape in the 5‐km buffers surrounding active leks went on to burn, and 96% of nests were in areas that burned. Post‐fire, only 54% of the land in the 5‐km buffers surrounding active leks had burned, indicating a shift in lek activity to overall more unburned areas. In 2018, only 22% of nests were in burned areas, but in 2019 this increased to 73% of nests in areas burned by the 2017 megafire. Nest densities in burned areas declined from 0.84/10 km^2^ ± 0.10 before the fire to 0.23/10 km^2^ ± 0.08 after the fire (Mann–Whitney *U*‐test = 151, *p* < .001).

After the fire, nest density in native working grasslands (0.34/10 km^2^ ± 0.08) was lower than before the fire (0.85/10 km^2^ ± 0.10; Mann–Whitney *U*‐test = 210, *p* < .001). Nest densities were greater in CRP fields (0.50/10 km^2^ ± 0.19) post‐fire, in strong contrast to before the fire, when no nests were in CRP fields. Nest density in CRP fields did not differ from nest density in native working grasslands post‐fire (Mann–Whitney *U*‐test = 531, *p* = .18). CRP fields made up a small portion of the landscape surrounding active leks both before (3.8%) and after (8.5%) the fire, but the percentage more than doubled post‐fire.

### 3.5 Chick survival

Before the fire, 14 broods (5 in 2014, 9 in 2015) were monitored for survival, and after the fire 7 (1 in 2018 and 6 in 2019) were monitored. Apparent survival of broods (number of broods with ≥1 chick surviving 35 days/total number of broods) was low before the fire (36%) with 5 broods consisting of 25 total chicks surviving to 35 days. After the fire, apparent survival of broods was greater (71%), but less chicks in each brood survived to 35 days, with 5 broods and 14 total chicks surviving to 35 days. The only competitive model of chick survival was the week‐specific survival model, indicating no difference in survival before and after the fire (Table 6). Overall estimated chick survival over 35 days was similar before (0.23 ± 0.07; 95% CI = 0.10–0.37) and after (0.27 ± 0.11; 95% CI = 0.05–0.49) the fire (Figure 2c). Mean detection probability was 0.81 ± 0.06 (95% CI = 0.67–0.89).

**TABLE 6:** Model selection of Lukacs' young survival models in Program MARK for lesser prairie‐chicken chick survival over 35‐days post‐hatch before (2014–2015) and after (2018–2019) the 2017 Starbuck fire in Clark County, Kansas, USA.

Model	K^a^	Δ AICc^b^	AICc^c^	*w*_*i*_ ^d^	Deviance^e^
Time	6	0.00	269.94	1.00	255.73
Time × Fire	11	13.22	283.16	0.00	253.16
Null	2	20.09	290.03	0.00	285.74
Fire	3	22.13	292.07	0.00	285.49

*Note*: Models tested include week‐specific survival (time), before or after fire (fire), a fire and time interaction, and null. Detection was held constant.

^a^ Number of parameters.

^b^ Difference in Akaike's Information Criterion, corrected for small sample size.

^c^ Akaike's Information Criterion, corrected for small sample size.

^d^ Akaike weights.

^e^ Deviance or −2 × loglikelihood.

## 4 DISCUSSION

While lesser prairie‐chickens persisted following the megafire, the population was negatively affected by megafire in the following three years. Lek counts indicated a reduced population size in the study area; however, adult, nest, and chick survival rates did not drastically differ after the fire. The strongest population effects observed were the abandonment of lek sites and previously inhabited areas, likely due to short‐term, widespread, habitat loss. Shifts in habitat use were largely related to movements to unburned areas (Parker, 2021), corroborating the importance of unburned islands as refugia for wildlife following wildfire (Steenvoorden et al., 2019). The large amount of high‐quality native working grassland in the area and presence of unburned CRP likely allowed persistence of this population and sustained survival rates (Robinson, Haukos, Plumb, Kraft, et al., 2018; Ross et al., 2016). Had the fire occurred where habitat was more limited, lesser prairie‐chickens may have been forced to use lower quality habitat in burned areas or embark on long‐distance dispersal movements, leading to lowered survival (Gulick, 2019; Johnson & Gaines, 1990; Yoder et al., 2004). Unlike species such as sage‐grouse that can experience reduced survival following wildfire (Anthony et al., 2022; Dudley et al., 2021; Foster et al., 2019), lesser prairie‐chickens may be resilient to megafire disturbance, possibly due to their evolutionary history in fire‐dependent grassland landscapes and contemporary occurrence in more fragmented grassland landscapes supplemented with CRP that provides refugia following disturbance.

### 4.1 Adult survival

Despite the extreme size and severity of the Starbuck fire, we did not observe a post‐fire change in survival of adult female lesser prairie‐chickens during either breeding or nonbreeding seasons. Previous estimates for breeding season survival in Kansas have been variable (0.39–0.76) depending on the year and site (Fields, 2004; Hagen et al., 2007; Plumb, 2015), but our estimates (before: 0.65, after: 0.61) were within and on the upper end of those bounds, even following megafire. Prior to the megafire, our study area had greater breeding season survival relative to five other study sites in Kansas and Colorado evaluated at the same time. Presumably greater survival estimates in the Clark County site pre‐fire was due to availability of large grasslands and well‐managed grazing that created quality habitat (Plumb, 2015; Sullins, 2017). Use of native working grasslands declined post‐fire but was augmented by increased use of CRP fields (Parker, 2021), potentially explaining why we did not observe a change in survival. Nonbreeding season survival estimates also did not differ following the fire. Estimates for nonbreeding survival have ranged from 0.66 to 0.86 in Kansas (Hagen et al., 2007; Robinson, Haukos, Plumb, Lautenbach, et al., 2018), with our post‐fire estimate (0.79) on the higher edge of this range. Continued high post‐fire survival rates were likely linked to the lack of mammalian predation events during the nonbreeding season post‐fire.

Predation is the primary cause of mortality for lesser prairie‐chickens (Boal, 2016); therefore, effects of the megafire on predator communities may have top‐down effects on lesser prairie‐chickens. We documented no mammalian predation events in the nonbreeding season post‐fire, but mammals were the main predators pre‐fire, and in previous studies in Kansas (Hagen et al., 2007; Plumb, 2015; Robinson, Haukos, Plumb, Lautenbach, et al., 2018). Coyotes and other mammalian predators often select for burned areas following fire (Ream, 1981; Ricketts, 2016; Thompson et al., 2008), which would suggest increased mammalian predation risk in our study post‐fire. However, in our study, lesser prairie‐chickens avoided burned areas and increased use of cropland and CRP post‐fire (Parker, 2021), which may have reduced predation risk by mammals if mammals were selecting burned areas post‐fire. Additionally, the shorter vegetation in burned areas following the fire could have promoted vigilance and escape from mammalian predators (Lautenbach et al., 2021; Lima, 1993; Winder et al., 2018). While overall predation decreased, raptor predation accounted for all nonbreeding season mortalities post‐fire. Similarly, in the tallgrass prairie, greater prairie‐chickens in recently burned and intensively grazed areas can have increased risk of avian predation (Winder et al., 2018). Increased raptor presence can occur following fire and is likely due to ease of detecting prey in more open areas (Bock & Bock, 1978). Shifts in predation patterns highlight the potential community and ecosystem‐level effects of megafire.

### 4.2 Nest survival and density

By integrating both nest survival and density, we were able to provide inference on both individual and population‐level reproductive effects of the megafire on lesser prairie‐chickens (Pidgeon et al., 2006). Of all demographic rates, nest survival was most affected by megafire (decreasing 49%) but did not differ at the 95% confidence interval. Among covariates we evaluated, visual obstruction best‐predicted nest survival, consistent with previous research (Grisham et al., 2014; Lautenbach et al., 2019; McNew et al., 2015). Our work demonstrates the key role of visual obstruction for lesser prairie‐chicken nest cover, and how fire may remove substantial thatch and residual cover needed by nesting lesser prairie‐chickens. A separate analysis of nest site vegetation found 100% visual obstruction pre‐fire ($\bar{x}$ = 1.91 dm) was nearly double post‐fire values ($\bar{x}$ = 1.01 dm), likely contributing to lower nest survival post‐fire (Parker, 2021). Megafire reduced multiple levels of visual obstruction (25%–100%) for several years post‐fire, but 100% visual obstruction was the only level selected post‐fire (Parker, 2021). Our estimates of the impact of visual obstruction on nest survival may be biased because we measured vegetation within 3 days of determining nest fate, which would allow vegetation to grow taller around successful nests (Gibson et al., 2016; Smith et al., 2018). However, *Tympanuchus* spp. largely rely on residual cover and dense thatch for reproduction measured at the 100% visual obstruction level and therefore may not change as much as at nests of other grouse species (Lautenbach et al., 2019). Overall nest survival analyses suggest lesser prairie‐chickens selected nest sites that maximize survival, but quality nest habitat availability was limited by lowered visual obstruction post‐fire.

Although weather‐related models were not informative based on our sample of nests, severe weather likely influenced nest survival, potentially confounding effects of the fire. The 2019 nesting season experienced record precipitation in our study area, with >27 cm of rain in May, compared to a long‐term May average of 8.6 cm (NOAA, 2020). Most females initiate and incubate nests in May and are particularly vulnerable to disturbance and predation (Pitman et al., 2006). During their study of greater prairie‐chickens in Oklahoma, USA, over the same time period, Londe et al. (2020, 2021) documented several female deaths on nests due to hail, as well as decreased nest success and an overall reproductive failure of their marked population due to extreme precipitation. While we did not observe hail mortality, it is possible heavy rain and hail forced hens to abandon nests that were later depredated.

Following the fire, we documented a 2.8× increase in the number of snake‐related nest depredations. Snakes can avoid fire through burrowing, and some studies have found increased population sizes several months post‐fire, but overall population response to fire is variable (Cavitt, 2000; Russell et al., 1999; Setser & Cavitt, 2003; Wilgers & Horne, 2006). Snakes may have increased predation of nests if alternative small mammal prey items were negatively affected by the intensity of the fire (Bock & Bock, 1978; Ream, 1981; Kaufman et al., 1990; Yarnell et al., 2007). In the tallgrass prairie, snake predation of grassland bird nests can increase in areas with denser vegetative cover (Jackrel & Reinart, 2009). Therefore, areas with more vegetation cover and concealment that promoted high nest density post‐fire may also have had increased snake abundance.

Relative nest densities fell 73% in burned areas post‐fire, which is not surprising given the fire reduced available nest habitat. Within the mixed‐grass prairie, lesser prairie‐chickens in a patch‐burn grazing system selected for nest sites in areas with the longest time since burn (≥4 years post‐fire; Lautenbach et al., 2021). Given such effects from small‐scale fire, it may be longer before nest habitat fully recovers following megafire. While vegetation monitoring and increased relative nest densities indicated recovery of nest habitat by 2019 (Parker et al., 2022), nest survival was the lowest estimated throughout the study in 2019. Low nest survival in 2019 was likely influenced by extreme weather events, but could also suggest nest habitat quality was not comparable to before the fire (Hagen et al., 2004; Lautenbach et al., 2019). We documented increased use of CRP for nesting post‐fire, likely because many CRP fields did not burn as they were surrounded by a matrix of cropland (Donovan et al., 2020) and can provide quality nest habitat (Sullins, Kraft, et al., 2018). The increased use of CRP post‐fire suggests CRP may be a refuge for lesser prairie‐chickens following megafire, similar to the increased use of CRP during drought (Sullins, Kraft, et al., 2018).

### 4.3 Chick survival

We found no difference in estimated chick survival before and after the fire. We expected chick survival to improve following megafire, as fire can create bare patches and increase forb cover, leading to quality brood habitat (Boyd & Bidwell, 2001). Instead, forb cover and brood habitat were temporarily reduced (Parker et al., 2022), likely contributing to no overall change in chick survival and small brood sizes. As lowered brood survival has been linked to extreme precipitation events during the primary brooding period from June–July, we expected lower survival given the greater amounts of summer precipitation post‐fire (Fields et al., 2006). This was the case in 2018, when the only monitored brood was killed by a hailstorm, resulting in an overall reproductive failure for our marked population that year. However, in 2019 the rain was concentrated in May, before the primary brooding period, likely minimizing any negative effects on brood survival. In fact, this may have aided brood survival because—when properly timed—large precipitation events can increase grasshopper and other arthropod populations necessary for chick nutrition (Branson, 2008; Sullins, Haukos, et al., 2018). We acknowledge the potential for bias in our methods because we used flush counts of unmarked chicks, which can obscure brood mixing and other sources of variation when estimating chick survival in other gallinaceous species (Dahlgren, Messmer, & Koons, 2010; Dahlgren, Messmer, Thacker, & Guttery, 2010; Kubečka et al., 2021; Orange et al., 2016). While such effects have not been thoroughly examined in lesser prairie‐chickens, previous work on VHF‐marked lesser prairie‐chicken chicks found no brood mixing or amalgamation (pers. comm D. A. Haukos). High chick and juvenile survival are key for lesser prairie‐chicken populations, particularly during periods of stress such as drought, and are, therefore, essential for post‐fire population recovery (Hagen et al., 2009; Ross et al., 2018). Unfortunately, even if future megafires improve brood habitat and survival, benefits are likely outweighed by negative effects of megafire on nesting habitat and survival (Boyd & Bidwell, 2001; Patten et al., 2007).

## 5 CONCLUSIONS

We documented no major changes in the survival parameters of lesser prairie‐chickens, but megafire reduced the population size in this area as evidenced by lowered lek counts post‐fire. While we did not monitor birds during, and in the spring and summer immediately following the 2017 fire, reproductive output was likely low given the fire's occurrence at the onset of the breeding season. The likely low reproductive output in 2017, followed by a complete reproductive failure in 2018 for our marked birds, makes it remarkable that lesser prairie‐chickens persisted in the area. Population persistence and relatively high adult and chick survival rates during our study underscore the resilience of lesser prairie‐chicken populations to megafire. Resilience was likely aided by large amounts of grassland in the area and unburned patches of CRP nearby that provided refuge (Ross et al., 2016; Steenvoorden et al., 2019; Sullins, Kraft, et al., 2018). Ample precipitation immediately and in the years following the fire likely helped sustain the population, as vegetation recovered relatively quickly (Parker et al., 2022). If drought conditions had occurred following the fire, the reduced population may not have survived, as lesser prairie‐chickens can be negatively affected by drought (Fritts et al., 2018; Grisham et al., 2013; Ross et al., 2016). In our study, lowered recruitment and lek counts indicate it will take >3 years for lesser prairie‐chicken populations to recover to pre‐fire conditions. Recovery will depend on local weather, grazing regimes, and other factors that contribute to the boom‐or‐bust population ecology of prairie‐chickens that fluctuates even when not affected by megafire (Hovick, Elmore, et al., 2015; Ross et al., 2018). Long‐term monitoring of populations following megafire will be necessary to determine population recovery times. If megafires remain infrequent, there may be positive long‐term effects for lesser prairie‐chickens in the form of increased plant diversity and reduction of invasive woody species (Ratajczak et al., 2012; Sirch et al., 2022; Twidwell et al., 2013, 2016). Given the importance of fire in the Great Plains and the unstable nature of lesser prairie‐chicken populations and habitat, working to control the size, intensity, and frequency of fires will be key in future lesser prairie‐chicken management.

## AUTHOR CONTRIBUTIONS

**Nicholas James Parker:** Conceptualization (equal); data curation (lead); formal analysis (lead); writing – original draft (lead); writing – review and editing (equal). **Daniel S. Sullins:** Conceptualization (equal); data curation (supporting); formal analysis (supporting); funding acquisition (equal); project administration (equal); supervision (equal); writing – review and editing (equal). **David Haukos:** Conceptualization (equal); data curation (supporting); funding acquisition (equal); project administration (equal); resources (equal); supervision (equal); writing – review and editing (equal). **Kent Fricke:** Funding acquisition (equal); project administration (equal); resources (equal); writing – review and editing (equal). **Christian Hagen:** Conceptualization (supporting); funding acquisition (equal); writing – review and editing (equal). **Adam A. Ahlers:** Conceptualization (supporting); writing – review and editing (equal).

## CONFLICT OF INTEREST

None declared.

## Supporting information


Appendix S1.

## Data Availability

Data have been archived in a Dryad repository: https://doi.org/10.5061/dryad.sj3tx9685.
