# Supplementary material for: Demographic effects of a megafire on a declining prairie grouse in the mixed‐grass prairie
Source: Ecol Evol. 2022 Nov 30;12(12):e9544. doi: 10.1002/ece3.9544 (PMC9712810; doi:10.1002/ece3.9544)
Supplement: Supplementary file 1 — Appendix S1. [file ECE3-12-e9544-s001.docx]

**Supplementary Text**

**Adult Predation**

Mammalian predation was identified by bite marks on transmitters, chewed leg bands, feathers matted with saliva, cached carcasses, and/or nearby tracks/scat. American badger (*Taxidea taxus*), coyote (*Canis latrans*), and swift fox (*Vulpes velox*) were all potential mammalian predators. Piles of plucked feathers, decapitated carcasses, removal of breast tissue, transmitters without tooth marks, and/or presence of avian scat were classified as avian predation. Potential avian predators in the area included red-tailed hawk (*Buteo jamaicensis*), ferruginous hawk (*B. regalis*), rough-legged hawk (*B. lagopus*), northern harrier (*Circus hudsonius*), and great-horned owl (*Bubo virginianus*). Mortalities with conflicting or lacking evidence, or on properties where we were denied permission, were labeled as unknown. We only used these three categories as there was no evidence of other known causes of lesser prairie-chicken mortality (e.g., collision with anthropogenic features, precipitation event, snake predation).

**Nest Predation**

Potential mammalian nest predators included coyote, badger, raccoon (*Procyon lotor*), striped skunk (*Mephitis mephitis*), thirteen-lined ground squirrels (*Ictidomys tridecemlineatus*), and other rodent and small mammal species. We classified empty and undisturbed nest bowls as snake predation; common species included gopher snake(*Pituophis catenifer*), prairie rattlesnake (*Crotalus virdis*), coachwhip (*Masticophis flagellum*), and eastern yellowbelly racer (*Coluber constrictor flaviventris)*. We categorized causes of unsuccessful nests with lacking or conflicting evidence as unknown.

**Management Implications**

Fire suppression will no longer be a viable option to prevent megafire, particularly when woody encroachment and climate change exacerbate the intensity of wildfires in the region (Donovan et al. 2020). We suggest implementation of prescribed burning and patch-burn grazing to reduce fuels and improve grassland health and lesser prairie-chicken habitat (Fuhlendorf and Engle 2001; Twidwell et al. 2013; Starns et al. 2019, 2020). Prescribed burns implemented at reasonable spatial (pasture-level) and temporal scales (every 4–10 years) would be most beneficial to preserve patches of key nesting habitat, while also creating quality forage and brood habitat (Lautenbach et al. 2021). Enrollment of CRP tracts should be maintained or targeted in areas with existing lesser prairie-chicken populations inhabiting grasslands with high fire risk. Following megafire, emergency haying and grazing of CRP may need to be limited for up to three years to maintain areas of lesser prairie-chicken habitat.

**Supplementary Tables**

Table S1. Monthly precipitation (cm) totals from 2014–2019 and 50-year (1970–2020) averages for Clark County, Kansas, USA (NOAA 2020).

| Month | Precipitation (cm) | | | | | | |
| --- | --- | --- | --- | --- | --- | --- | --- |
|  | 2014 | 2015 | 2016 | 2017 | 2018 | 2019 | Average |
| Jan | 0.48 | 1.63 | 0.76 | 6.99 | 0.05 | 1.68 | 1.55 |
| Feb | 1.88 | 2.26 | 0.84 | 0.18 | 0.23 | 2.08 | 1.75 |
| Mar | 1.02 | 1.65 | 0.58 | 6.73 | 1.35 | 5.08 | 4.29 |
| Apr | 1.12 | 7.52 | 15.34 | 12.60 | 3.02 | 1.12 | 5.18 |
| May | 2.06 | 21.95 | 6.96 | 6.50 | 10.59 | 27.31 | 8.61 |
| Jun | 21.36 | 7.44 | 8.36 | 5.18 | 12.45 | 9.58 | 8.61 |
| Jul | 11.53 | 12.93 | 12.09 | 4.60 | 11.46 | 4.57 | 7.39 |
| Aug | 5.36 | 6.25 | 8.08 | 5.59 | 12.57 | 9.04 | 7.09 |
| Sep | 3.40 | 3.76 | 4.88 | 5.31 | 7.39 | 1.83 | 4.90 |
| Oct | 5.28 | 6.91 | 0.86 | 2.24 | 10.77 | 3.84 | 4.50 |
| Nov | 0.20 | 4.62 | 1.17 | 0.08 | 0.76 | 0.74 | 2.31 |
| Dec | 4.24 | 4.52 | 1.55 | 0.00 | 2.57 | 3.20 | 2.08 |
|  |  |  |  |  |  |  |  |
| Total | 57.94 | 81.43 | 61.47 | 55.98 | 73.20 | 70.05 | 58.27 |

Table S2. High counts of male attendance at leks in Clark County, Kansas, USA, surveyed before (2014–2015) and after (2018–2019) the Starbuck fire in March 2017. Leks were surveyed from 15 Mar–1 May before 1000 and under favorable weather conditions.

| **Lek** | **2014** | **2015** | **2018** | **2019** | **Within Burned Area** |
| --- | --- | --- | --- | --- | --- |
| 1 | 3 | 0 | 0 | 0 | Yes |
| 2 | 24 | 19 | 0 | 0 | Yes |
| 3 | 3 | 4 | 0 | 0 | Yes |
| 4 | 7 | 4 | 4 | 2 | Yes |
| 5 | 15 | 15 | 0 | 0 | Yes |
| 6 | 14 | 10 | 2 | 1 | Yes |
| 7 | 3 | 8 | 2 | 0 | Yes |
| 8 | 13 | 13 | 0 | 0 | Yes |
| 9 | 14 | 9 | 0 | 0 | Yes |
| 10 | 15 | 20 | 19 | 12 | Yes |
| 11 | 12 | 17 | 9 | 5 | Yes |
| 12 | 5 | 11 | 11 | 12 | No |
| 13 | NA^a^ | 22 | 4 | 11 | Yes |
| 14 | NA^a^ | 7 | 1 | 0 | Yes |
| 15 | NA^a^ | 3 | 0 | 0 | Yes |
| **Total** | **128** | **162** | **52** | **43** |  |

^a^Lek not surveyed or found until 2015.

Table S3. Model selection results testing nest survival in relation to nest vegetation characteristics for lesser prairie-chickens in Clark County, Kansas, USA. Models included single and quadratic variable combinations of 0–100% visual obstruction (VOR), litter depth, standard deviation of litter depth, percent cover of different functional groups (bare ground, shrub, forb, grass, litter), and null (intercept only).

| **Model** | **K^a^** | **Δ AICc^b^** | **AICc^c^** | ***w_i_*^d^** | **Deviance^e^** |
| --- | --- | --- | --- | --- | --- |
| 100% VOR | 2 | 0.00 | 400.44 | 0.24 | 396.43 |
| 75% VOR | 2 | 0.47 | 400.92 | 0.19 | 396.91 |
| 25% VOR | 2 | 0.96 | 401.41 | 0.15 | 397.40 |
| 50% VOR | 2 | 1.22 | 401.66 | 0.13 | 397.65 |
| 100% VOR^2^ | 3 | 1.98 | 402.42 | 0.09 | 396.40 |
| 75% VOR^2^ | 3 | 2.46 | 402.90 | 0.07 | 396.88 |
| 25% VOR^2^ | 3 | 2.97 | 403.41 | 0.05 | 397.39 |
| 50% VOR^2^ | 3 | 3.14 | 403.59 | 0.05 | 397.57 |
| 0% VOR | 2 | 5.45 | 405.89 | 0.02 | 401.88 |
| 0% VOR^2^ | 3 | 7.35 | 407.80 | 0.01 | 401.78 |
| Litter Depth | 2 | 12.22 | 412.67 | 0.00 | 408.66 |
| Vegetation Height | 2 | 13.11 | 413.55 | 0.00 | 409.54 |
| Litter Depth^2^ | 3 | 14.14 | 414.58 | 0.00 | 408.56 |
| Null | 1 | 15.79 | 416.24 | 0.00 | 414.23 |
| Shrub | 2 | 17.57 | 418.02 | 0.00 | 414.01 |
| Forb | 2 | 17.79 | 418.23 | 0.00 | 414.22 |
| Bare | 2 | 17.79 | 418.24 | 0.00 | 414.23 |
| Litter | 2 | 17.80 | 418.24 | 0.00 | 414.23 |
| Grass | 2 | 17.80 | 418.24 | 0.00 | 414.23 |

^a^Number of parameters.

^b^Difference in Akaike’s Information Criterion, corrected for small sample size.

^c^Akaike’s Information Criterion, corrected for small sample size.

^d^Akaike weights.

^e^Deviance or -2*loglikelihood.
